# Maternal psychiatric disease and epigenetic evidence suggest a common biology for poor fetal growth

**DOI:** 10.1186/s12884-015-0627-8

**Published:** 2015-08-25

**Authors:** Timothy H. Ciesielski, Carmen J. Marsit, Scott M. Williams

**Affiliations:** 1 Department of Pharmacology and Toxicology, Geisel School of Medicine at Dartmouth, Hanover, NH USA; 2 Department of Epidemiology, Geisel School of Medicine at Dartmouth, Hanover, NH USA; 3 Institute for Quantitative Biomedical Sciences, Dartmouth College, Hanover, NH USA; 4 Department of Genetics, Geisel School of Medicine at Dartmouth, Hanover, NH USA

## Abstract

**Background:**

We sought to identify and characterize predictors of poor fetal growth among variables extracted from perinatal medical records to gain insight into potential etiologic mechanisms. In this process we reevaluated a previously observed association between poor fetal growth and maternal psychiatric disease.

**Methods:**

We evaluated 449 deliveries of >36 weeks gestation that occurred between 9/2008 and 9/2010 at the Women and Infants Hospital in Providence Rhode Island. This study group was oversampled for Small-for-Gestational-Age (SGA) infants and excluded Large-for-Gestational-Age (LGA) infants. We assessed the associations between recorded clinical variables and impaired fetal growth: SGA or Intrauterine Growth Restriction (IUGR) diagnosis. After validating the previously observed association between maternal psychiatric disease and impaired fetal growth we addressed weaknesses in the prior studies by explicitly considering antidepressant use and the timing of symptoms with respect to pregnancy. We then evaluated DNA methylation levels at 27 candidate loci in placenta from a subset of these deliveries (n = 197) to examine if epigenetic variation could provide insight into the mechanisms that cause this co-morbidity.

**Results:**

Infants of mothers with prenatal psychiatric disease (Depression, Anxiety, OCD/Panic) had increased odds of poor fetal growth (OR_adjusted_ = 3.36, 95%CI: 1.38-8.14). This relationship was similar among those who were treated with antidepressants (OR_adjusted_ = 3.69, 95%CI: 1.31-10.45) and among those who were not (OR_adjusted_ = 3.19, 95%CI: 1.30-7.83). Among those with a history of psychiatric disease but no active disease in pregnancy the OR_adjusted_ was 0.45 (95%CI: 0.09-2.35). A locus near the transcription start site of the leptin receptor (cg21655790) had methylation levels that were decreased in the presence of: 1) SGA/IUGR, and 2) active but not resolved psychiatric disease (among mothers not on antidepressants).

**Conclusions:**

These results validate and further characterize the association between maternal psychiatric disease and poor fetal growth. Because the association appears to depend on active psychiatric disease, this suggests a transient and potentially modifiable pathophysiology. The molecular findings in this study suggest that altered leptin signaling may be involved in the biological mechanisms that link prenatal maternal psychiatric symptoms and poor fetal growth.

**Electronic supplementary material:**

The online version of this article (doi:10.1186/s12884-015-0627-8) contains supplementary material, which is available to authorized users.

## Background

Several epidemiologic studies have evaluated links between antenatal maternal psychiatric disease and poor fetal growth. While the majority of reports have focused on depression (reviewed in [1–3]), other conditions such as bipolar disorder [4, 5], panic disorder [6, 7], and mental illness as a whole [8] have also been assessed. Unfortunately, as a group, the existing studies of maternal psychiatric disease and poor fetal growth are difficult to interpret because of ambiguous or non-comparable fetal growth outcomes (e.g. Low-Birth-Weight [LBW] vs. Small-for-Gestational-Age [SGA]) and inconsistent findings. It is also largely unclear if poor fetal growth is associated with the experience of psychiatric symptoms during pregnancy or other characteristics of mothers with psychiatric diagnoses whether they are symptomatic in pregnancy or not. An additional complication is that observational studies are limited in their ability to separate associations with psychiatric disease from associations with psychiatric medication. Despite these difficulties, evaluating associations with clinical psychiatric diagnoses is important because other psychosocial variables that have been associated with poor fetal growth [9–11] may not be available in medical records, and thus have limited potential to inform obstetrical care.

Identifying predictors of poor fetal growth is key to finding etiologic subtypes and defining homogeneous groups for pathophysiologic study. Perinatal medical records can provide a rich source of potential predictors for distinguishing these subtypes. In this study we analyzed a sample of 449 mother-infant pairs to identify and characterize clinical predictors of poor fetal growth, and in this process we re-evaluated the association between prenatal maternal psychiatric disease and poor fetal growth. These analyses address some weaknesses in the prior studies by utilizing medically interpretable outcomes (SGA and Intrauterine Growth Retardation [IUGR]), considering the timing of psychiatric diagnosis with respect to pregnancy, and evaluating antidepressant treated and untreated mothers separately. We also present an exploratory analysis of placental DNA methylation patterns to examine biological factors that may be involved in the pathophysiology linking maternal psychiatric symptoms to poor fetal growth.

## Methods

### Data source and study group

A structured form was used to abstract perinatal clinical information from the medical records of 490 deliveries that occurred between September 2008 and September 2010 at the Women and Infants Hospital in Providence Rhode Island. Eligible deliveries were from pregnancies of ≥36 weeks gestation that resulted in viable infants and were not complicated by diagnosed preeclampsia or known fetal genetic conditions.

The primary objective of this parent cohort was to examine how epigenetic features of the placenta were related to growth restriction. In order to improve power and efficiency, infants considered small for gestational age were over-represented within the sample. Infants not considered SGA were then selected to match the SGA infants on sex, maternal age (within 3 years), and gestational age (within 1 week). The study procedures were approved by the Institutional Review Boards of the Women and Infants Hospital, Brown University, and the Committee for Protection of Human Subjects at Dartmouth College. A waiver of consent was obtained through approval of the Women and Infants Hospital IRB. Samples and data were collected in a de-identified fashion.

For the analysis, SGA was defined as <10th percentile for birth weight based on the 2003 Fenton infant growth chart [12] and IUGR was as noted in the medical records (typically: sonographic measurements estimating <10th percentile for weight based on gestational age [13]). Poor fetal growth was defined as the presence of SGA, IUGR, or both. Large for Gestational Age (LGA, >90^th^ percentile for birth weight) deliveries were excluded from the analyses (n = 41), leaving a study group of 449 deliveries. This clarified the comparisons being made by including only AGA infants in the reference group (i.e. we were interested in comparing SGA and AGA infants, not SGA and a mixture of AGA and LGA infants). Maternal psychiatric status was recorded as the presence of Depression, Anxiety, or OCD/Panic disorder in pregnancy and history of Depression, Anxiety, or OCD/Panic disorder at any time (not necessarily during pregnancy).

### DNA methylation data

Epigenetic analyses were conducted using placenta from a subset of deliveries (n = 197). Placental samples were obtained within 2 h of delivery, and DNA methylation levels were measured using the Human-Methylation-27 BeadChip array (Illumina Inc., San Diego, CA). Details of these protocols have been previously described [14].

### Statistical analyses

Logistic Regression was used to determine the unadjusted associations between the clinical variables and SGA/IUGR status. Differences in the distributions of clinical and outcome variables (SGA/IUGR) by maternal psychiatric status were determined by Wilcoxon-Mann–Whitney, Chi-square, or Fisher’s exact tests as appropriate. Crude and adjusted logistic regression models were used to estimate the association between maternal psychiatric disease in pregnancy and poor fetal growth (SGA/IUGR). Models were adjusted for variables that may confound the relationship between maternal psychiatric disease and poor fetal growth based on subject matter knowledge.

We evaluated for differences in the distribution of participant characteristics between those with placental methylation data and those without using Wilcoxon-Mann–Whitney, Chi-Square, and Fisher’s Exact tests as appropriate (Additional file 1: Table S12). Candidate methylation loci were identified through a literature and locus annotation search described in the supplemental materials (Additional file 1). Briefly: We identified a recent paper that listed genes linked to poor fetal growth [15]. These genes were often implicated by association with variants within the gene or their differential expression patterns. Subsequent investigation of these genes lead to the identification of other genes linked to SGA/IUGR, and the hits from Banister et al. [14] were also evaluated. We then scanned OMIM (http://www.ncbi.nlm.nih.gov/omim) and Pubmed (http://www.ncbi.nlm.nih.gov/pubmed/) for evidence linking these genes to psychiatric disease. This process identified 11 genes with links to both SGA/IUGR and psychiatric disease (Additional file 1: Table S3). One imprinted gene, *MEG3* (Maternally Expressed Gene 3) [16, 17], was excluded because methylation measurements at imprinted genes could be unreliable on the Illumina 27 K array platform, as the targeted loci may not be in the imprinting control region. Then we performed texts searches for these 10 genes in the annotation file for the Human-Methylation-27 BeadChip array (Illumina Inc., San Diego, CA) and identified 27 loci with annotations that related to these genes (Additional file 1: Table S4).

Logistic regression was used to determine if any of these 27 loci had methylation patterns that were significantly associated with SGA/IUGR or psychiatric diagnosis (false discovery rate [FDR] adjusted p-value < 0.05). For the loci with methylation patterns that differed by SGA/IUGR status, Fisher’s exact tests (FDR adjusted) were used to determine if methylation status (above vs. below median) differed between these three categories: 1) no psychiatric diagnosis and no antidepressant, 2) psychiatric diagnosis and no antidepressant, 3) psychiatric diagnosis and antidepressant). Exact binomial tests within these 3 strata (FDR adjusted) were conducted to determine if any of the strata had a significant excess of above or below median methylation values. After excluding women on antidepressants, a similar approach was used to evaluate associations separately among those with active psychiatric disease, resolved psychiatric disease, and no history of psychiatric disease. We also evaluated if other measured factors differed between those with no psychiatric disease, un-medicated psychiatric disease and medicated psychiatric disease (Kruskal-Wallis or Fisher’s Exact tests as appropriate, Additional file 1: Table S8). Mean methylation levels were also calculated (Additional file 1: Table S6 and S7). Analyses were conducted using SAS 9.1.3 (SAS Institute Inc., Cary, NC).

## Results

Characteristics of the study group are presented in Table 1. The study group was oversampled for SGA deliveries and excluded LGA deliveries. 155 out of the 449 (34.5 %) had evidence of impaired fetal growth (SGA or IUGR diagnosis or both). Infant sex was evenly divided (50.3 % male, 49.7 % female), and the mothers represent a group that is oversampled for non-white race compared the Rhode Island population in general based on the 2010 census [18].

**Table 1:** Characteristics of the 449 mother-infant pairs in the study group

	Median	IQR
Maternal age in years	28	24 - 32
Maternal height in cm (98 missing)	160	157 - 165
Maternal weight in kg (93 missing)	63.3	55.5 - 75.0
Maternal BMI (104 missing)	24.6	21.7 - 28.9
	Frequency	%
Race		
White	260	57.9
Black	47	10.5
Asian	27	6.0
Other race or unknown	115	25.6
Hypertension in pregnancy (2 missing)		
No	420	94.0
Yes	27	6.0
Diabetes in pregnancy (2 missing)		
No	430	96.2
Yes	17	3.8
Tobacco in pregnancy (2 missing)		
No	399	89.3
Yes	48	10.7
Alcohol in pregnancy (1 missing)		
No	445	99.3
Yes	3	0.7
Recreational drugs in pregnancy (3 missing)		
No	436	97.8
Yes	10	2.2
Prenatal vitamins (7 missing)		
No	87	19.7
Yes	355	80.3
Insurance		
Public	205	45.7
Private	236	52.6
Military	1	0.2
Self-Pay	7	1.6
Antidepressant medication in pregnancy (27 missing)		
No	401	95.0
Yes	21	5.0
Psychiatric diagnosis in pregnancy^a^ (10 missing)		
No	381	86.8
Yes	58	13.2
First pregnancy (3 missing)		
No	284	63.7
Yes	162	36.3
Maternal age categories (in years)		
18-19	30	6.7
20-34	342	76.2
35-43	77	17.2
Infant sex		
Male	226	50.3
Female	223	49.7

^a^Depression, Anxiety, or OCD/Panic disorder in pregnancy

In unadjusted logistic regression analyses, prenatal maternal depression (OR = 3.90, 95 % CI: 2.06-7.36, n = 46), anxiety (OR = 3.15, 95 % CI: 1.51-6.57, n = 32), and OCD/Panic disorders (OR = 8.23, 95 % CI: 1.73-39.26, n = 10) were associated with poor fetal growth, as was prenatal maternal psychiatric disease as a whole (depression, anxiety, or OCD/Panic, OR = 3.93, 95 % CI: 2.21-6.98, n = 58). Logistic regression analyses for additional clinical variables are presented in Additional file 1: Table S1. The associations with these prenatal psychiatric variables remained significant after accounting for multiple testing (FDR adjusted p-values, prenatal depression: 6.7 × 10^−04^, prenatal anxiety: 1.1 × 10^−02^, prenatal OCD/Panic: 3.0 × 10^−02^, and prenatal psychiatric disease: 1.4 × 10^−04^) and the p-values for prenatal depression and prenatal psychiatric disease were the two smallest in Additional file 1: Table S1. Of participants who had a psychiatric diagnosis in pregnancy, 62 % (36/58) had poor fetal growth, while 29 % (112/381) of the remaining participants had poor fetal growth. Women with prenatal psychiatric diagnoses were more likely to use tobacco and antidepressant medication, and they also differed with respect to insurance type, a crude measure of socioeconomic status (Fisher’s exact tests p < 0.05). 64 % of women with prenatal psychiatric diagnoses had public insurance compared to 43 % of women without prenatal psychiatric diagnoses. Additional information on covariate distributions by maternal psychiatric status are available in Additional file 1: Table S2.

The association between maternal psychiatric diagnosis in pregnancy and poor fetal growth remained significant after adjusting for: antidepressant medication use, tobacco use, alcohol use, recreational drug use, first pregnancy vs. not first pregnancy, race (white, black, Asian, or other race/unknown), insurance (public, private, military, self-pay), maternal age (<20, 20–34, and >34 years), maternal height, and maternal weight (OR_adjusted_ = 3.36, 95 % CI: 1.38-8.14, Table 2). Adjusted ORs for poor fetal growth were similar among the specific diagnoses evaluated here: Depression, Anxiety, and OCD/Panic (Table 3). The odds of poor fetal growth were increased with psychiatric diagnosis regardless of whether the mother was on antidepressant medication (OR_adjusted_ = 3.69, 95 % CI: 1.31-10.45) or not (OR_adjusted_ = 3.19, 95 % CI: 1.30-7.83, Table 4). Among women with a history of psychiatric diagnosis, the odds of poor fetal growth were only increased only if they had active disease in pregnancy (active disease OR_adjusted_ = 2.87, 95 % CI: 1.15-7.20, resolved disease OR_adjusted_ = 0.45, 95 % CI: 0.09-2.35, Table 5).

**Table 2:** Odds Ratios for poor fetal growth associated with active maternal psychiatric diagnosis

	OR	95 % CI	p-value
Unadjusted	3.93	2.21–6.98	3.0 × 10^−06^
Adjusted^a^	3.36	1.38–8.14	7.4 × 10^−03^

^a^Adjusted for antidepressant medication, tobacco use, alcohol use, recreational drug use, first pregnancy vs. not first pregnancy), race (white, black, Asian, or other race/unknown), insurance (public, private, military, self-pay), maternal age (<20, 20–34, and >34 years), maternal height, and maternal weight

**Table 3:** Odds Ratios for poor fetal growth by specific maternal psychiatric diagnoses

	OR	95 % CI	p-value
Depression in pregnancy (n = 46)			
Unadjusted	3.90	2.06–7.36	2.8 × 10^−05^
Adjusted^a^	2.86	1.02–8.05	4.6 × 10^−02^
Anxiety in pregnancy (n = 32)			
Unadjusted	3.15	1.51–6.57	2.2 × 10^−03^
Adjusted^a^	2.94	1.09–7.97	3.4 × 10^−02^
OCD or panic disorder in pregnancy (n =10)^b^			
Unadjusted	8.23	1.73–39.26	8.2 × 10^−03^
Adjusted^a^	2.74	0.46–16.49	2.7 × 10^−01^

^a^Adjusted for antidepressant medication, tobacco use, alcohol use, recreational drug use, first pregnancy vs. not first pregnancy, race (white, black, Asian, or other race/unknown), insurance (public, private, military, self-pay), maternal age (<20, 20–34, and >34 years), maternal height, and maternal weight

^b^The effect estimate precision in this category is very low due to a small n, but the direction of association is consistent with the other two diagnoses

**Table 4:** Odds Ratios for poor fetal growth associated with medicated and un-medicated maternal psychiatric diagnosis

	OR^a^	95 % CI	p-value
Unadjusted			
Psychiatric diagnosis with no antidepressant use (n =36)	4.05	2.00–8.24	1.1 × 10^−04^
Psychiatric diagnosis with antidepressant use (n =19)	4.42	1.69–11.56	2.4 × 10^−03^
Adjusted^b^			
Psychiatric diagnosis with no antidepressant use	3.19	1.30–7.83	1.1 × 10^−02^
Psychiatric diagnosis with antidepressant use	3.69	1.31–10.45	1.4 × 10^−02^

^a^Reference is those with no psychiatric diagnosis and no antidepressant medication use (n =358). Note: there was 1 participant on antidepressant medication with no reported psychiatric diagnosis in pregnancy but this group was too small to evaluate here

^b^Adjusted for tobacco use, alcohol use, recreational drug use, first pregnancy vs. not first pregnancy, race (white, black, asian, or other race/unknown), insurance (public, private, military, self-pay), maternal age (<20, 20–34, and >34 years), maternal height, and maternal weight

**Table 5:** Odds Ratios for poor fetal growth by the timing of maternal psychiatric diagnosis

	OR^a^	95 % CI	p-value
Unadjusted			
History of psychiatric diagnosis but no active diagnosis during pregnancy (n = 18)	0.93	0.32–2.67	8.9 × 10^−01^
History of Psychiatric diagnosis and active diagnosis during pregnancy (n = 56)	3.73	2.09–6.68	9.2 × 10^−06^
Adjusted^b^			
History of Psychiatric diagnosis but no active diagnosis during pregnancy (n = 10)	0.45	0.09–2.35	3.4 × 10^−01^
History of Psychiatric diagnosis and active diagnosis during pregnancy (n = 42)	2.87	1.15–7.20	2.4 × 10^−02^

^a^Reference is those with no recorded history of ever having Depression, Anxiety, or OCD/Panic disorder (n = 362). Note: there were 2 people with newly reported psychiatric diagnosis in pregnancy and no prior history of psychiatric diagnosis but this group was too small to evaluate here

^b^Adjusted for antidepressant medication, tobacco use, alcohol use, recreational drug use, first pregnancy vs. not first pregnancy, race (white, black, asian, or other race/unknown), insurance (public, private, military, self-pay), maternal age (<20, 20–34, and >34 years), maternal height, and maternal weight

DNA methylation analyses using the Illumina Infinium 27 K array were performed on placenta from a subset of 197 participants. In this subset there were 89 subjects with SGA/IUGR and 26 with psychiatric diagnosis. Those included in the methylation analyses were similar to those excluded from the methylation analyses (Additional file 1: Table S12) but the number of previous preterm births was slightly higher among those included (mean: 0.20, standard deviation: 0.61 vs. mean: 0.10, standard deviation: 0.35; Wilcoxon test p = 5.0x10^−02^). When examining 27 candidate loci from 15 genes, 6 loci had placental DNA methylation levels that were significantly associated with SGA/IUGR status but no loci had methylation levels that were significantly associated with psychiatric status (FDR adjusted p-values <0.05). These 6 loci were located near the transcription start sites of 5 genes: *FKBP5*, *RPE65*, *LEPR*, *LEPROTL1*, *NFKBIZ* (2 methylation sites for *NFKBIZ*). Odds of poor fetal growth increased with higher methylation at *FKBP5*/cg08636224 and *RPE65*/cg11724759 and decreased with higher methylation at *LEPR/*cg21655790, *LEPROTL1*/cg00177923, and *NFKBIZ* /cg26284390/cg15006396 (details in Additional file 1: Table S5).

To assess if antidepressant use might be masking relationships between psychiatric disease and methylation status we evaluated methylation levels within strata defined by both psychiatric diagnosis and antidepressant medication use (i.e. no psychiatric diagnosis, psychiatric diagnosis on antidepressants, and psychiatric diagnosis not on antidepressants). Given the low numbers of participants in some of these strata, we used Fisher’s exact tests to determine if methylation status (above vs. below median) differed between the three groups for each of the 6 loci (Table 6; mean methylation levels are presented in Supplemental Table 6 in Additional file 1). These analyses revealed that methylation at *LEPR*/cg21655790, a locus near the transcription start site of the leptin receptor, differed between the three strata (Fisher’s exact test: FDR adjusted p = 0.029). Three follow-up exact binomial tests revealed that those with psychiatric diagnosis and no antidepressant medication were more likely to have below median placental methylation at *LEPR*/cg21655790 (FDR adjusted p = 0.01). Furthermore, among those not on antidepressants, placental methylation at this locus differed between the following 3 groups: 1) those with no history of psychiatric disease, 2) those with a history but no active disease, and 3) those with a history and active disease (Table 7, Fisher’s exact test p = 4.8 × 10^−4^, mean methylation levels are presented in Supplemental Table 7 in Additional file 1). Follow-up exact binomial tests in these three groups revealed that only those with active psychiatric diagnosis were more likely to have below median placental methylation at *LEPR*/cg21655790 (FDR adjusted p = 0.01).

**Table 6:** Methylation at 6 candidate loci within strata defined by psychiatric and medication status

Gene and locus	Methylation (adjusted Beta)	No psychiatric diagnosis and no antidepressant (n = 159)	Psychiatric diagnosis and no antidepressant (n = 13)	Psychiatric diagnosis and antidepressant (n = 12)	FDR adjusted p-value (Fisher’s exact test)
		59/159 (37.1 %)	10/13 (76.9 %)	9/12 (75.0 %)	
		had SGA or IUGR	had SGA or IUGR	had SGA or IUGR	
FKBP5	<= Median	79 (49.7 %)	4 (30.8 %)	8 (66.7 %)	3.7 × 10^−01^
cg08636224	> Median	80 (50.3 %)	9 (69.2 %)	4 (33.3 %)	
RPE65	<= Median	85 (53.5 %)	4 (30.8 %)	6 (50.0 %)	3.7 × 10^−01^
cg11724759	> Median	74 (46.5 %)	9 (69.2 %)	6 (50.0 %)	
LEPR^a^	<= Median	76 (47.8 %)	12 (92.3 %)	5 (41.7 %)	2.9 × 10^−02^
cg21655790	> Median	83 (52.2 %)	1 (7.7 %)	7 (58.3 %)	
LEPROTL1	<= Median	80 (50.3 %)	6 (46.2 %)	3 (25.0 %)	3.7 × 10^−01^
cg00177923	> Median	79 (49.7 %)	7 (53.9 %)	9 (75.0 %)	
NFKBIZ	<= Median	76 (47.8 %)	11 (84.6 %)	6 (50.0 %)	1.1 × 10^−01^
cg26284390	> Median	83 (52.2 %)	2 (15.4 %)	6 (50.0 %)	
NFKBIZ	<= Median	80 (50.3 %)	8 (61.5 %)	5 (41.7 %)	6.5 × 10^−01^
cg15006396	> Median	79 (49.7 %)	5 (38.5 %)	7 (58.3 %)	

^a^Methylation at LEPR/cg21655790 differs significantly between these three categories (Fisher’s exact test: FDR adjusted p = 0.029). Three follow up tests (exact binomial tests that p = 0.5) revealed that those with psychiatric diagnosis and no antidepressant medication are more likely to have below median placental methylation at LEPR/cg21655790 (FDR adjusted p = 0.01). NFKBIZ/cg26284390 has a very similar pattern of results as LEPR/cg21655790 but it misses significance in the Fisher’s exact test (FDR adjusted p = 0.107)

**Table 7:** Methylation at the leptin receptor locus by timing of psychiatric diagnosis among those not on antidepressant medication

Gene and locus	Methylation (adjusted Beta)	No History of Psychiatric Diagnosis and no Active Psychiatric Diagnosis^b^ (n = 151)	History of Psychiatric Diagnosis but no Active Psychiatric Diagnosis (n = 8)	History of Psychiatric Diagnosis and Active Psychiatric Diagnosis (n = 13)	p-value (Fisher’s exact test)
		57/151 (37.8 %)	2/8 (25.0 %)	10/13 (76.9 %)	
		had SGA or IUGR	had SGA or IUGR	had SGA or IUGR	
LEPR^a^	<= Median	75 (49.7 %)	1 (12.5 %)	12 (92.3 %)	4.8 × 10^−4^
cg21655790	> Median	76 (50.3 %)	7 (87.5 %)	1 (7.7 %)	

^a^Methylation differs between these three categories (Fisher’s exact test p = 4.8 x 10^−4^). Three follow up tests (exact binomial tests that p = 0.5) revealed that those with active maternal psychiatric diagnoses are more likely to have below median placental methylation at LEPR/cg21655790 (FDR adjusted p = 0.01)

^b^As noted in Table 5, there were 2 people with no prior history of psychiatric diagnosis who had newly reported psychiatric diagnosis in pregnancy. Neither of these participants had LEPR/cg2165570 methylation data

We also evaluated if other factors differed between the 3 psychiatric status categories from Table S7, to identify if additional measured factors were related to maternal psychiatric disease (Additional file 1: Table S8). Prenatal tobacco use was associated with active un-medicated psychiatric disease (Fisher’s Exact test p = 3.0 × 10^−3^), and also demonstrated a non-significant relationship with below median placental methylation at LEPR/cg21655790 (Fisher’s exact test p = 1.3 × 10^−1^ see Additional file 1: Table S9). The association between un-medicated psychiatric disease and decreased methylation at LEPR/cg21655790 is still significant when those with prenatal tobacco use are excluded from the analysis (Fisher’s exact test: p = 3.3 × 10^−2^, Additional file 1: Table S10), and the association between decreased methylation at LEPR/cg21655790 and poor fetal growth still significant in a logistic regression model adjusted for prenatal tobacco use (p = 8.5 × 10^−3^, Additional file 1: Table S11).

## Discussion

Our analyses validate and further characterize previously observed associations [1, 3, 6, 8] between maternal psychiatric disease and poor fetal growth. In these data, mothers with evidence of prenatal psychiatric disease (Depression, Anxiety, OCD/Panic) had increased odds of having a pregnancy with poor fetal growth (IUGR/SGA). This relationship was similar regardless of whether the women were treated with antidepressants or not. The findings among antidepressant treated mothers are difficult to interpret because it is hard to separate associations with medication use from associations with psychiatric disease. Additionally, a direct comparison of the ORs from the antidepressant treated and untreated groups is not very informative because medication was not allocated randomly (e.g. those on antidepressants may have had more severe disease). However the results among untreated mothers, are not affected by these complications, and they suggest an independent association between psychiatric disease and poor fetal growth. We did not detect any evidence for an association, or even a similar trend, among women with resolved disease, but we had limited power to do so. Taken together these results suggest that the association is driven by transient and thus potentially modifiable factors.

The remaining analyses explored DNA methylation patterns in placentae from a subset of these deliveries to identify biological factors that could be involved in the pathophysiology that links maternal psychiatric disease and poor fetal growth. The results of the initial epidemiologic analyses were used to guide these exploratory analyses because they led to several predictions about the features that etiologically relevant methylation patterns might have. If placental methylation at any locus was involved in this shared pathophysiology then methylation levels at this locus should be associated with both poor fetal growth and maternal psychiatric disease, and the direction of association should be the same for both phenotypes. In addition, the association should be present in active but not resolved disease. One of the 27 loci, LEPR/cg21655790, had methylation patterns that were consistent with all of these predictions. This suggests that decreased placental DNA methylation near the leptin receptor locus (cg21655790) may be involved with the biological processes that result in poor fetal growth in women with active psychiatric disease.

We were also able to strengthen our findings by conducting additional analyses to explore alternative explanations for this pattern in the methylation data (Additional file 1: Tables S8–S11). In these additional analyses we found that prenatal tobacco use was significantly associated with active psychiatric disease and was non-significantly associated with decreased methylation at LEPR/cg21655790. However, after accounting for prenatal tobacco use, the associations between LEPR/cg21655790 methylation and 1) un-medicated psychiatric disease, and 2) poor fetal growth were both still significant. Furthermore, the association between active psychiatric disease and poor fetal growth was still significant after adjusting for prenatal tobacco use. In sum, even though tobacco use frequently occurs with prenatal psychiatric disease, the links between active psychiatric disease, poor fetal growth, and decreased methylation at the LEPR locus are not explained by prenatal tobacco use.

Leptin is an endocrine signaling molecule that has an established role in regulating energy use and nutritional intake [19], but it appears to also play a role in the pathophysiology of depression and anxiety. Observational studies in humans suggest that depression is associated with decreased circulating leptin levels (reviewed briefly in [20, 21]), however, there have been some inconsistencies between the studies [22–28]. It has been suggested that complicating factors such as leptin sensitivity may help to explain these discrepancies and that leptin levels may only be decreased in a subset of depressed patients [20, 21]. Defining and characterizing this putative subgroup may help to advance our understanding of the association between depression and poor fetal growth. In addition to these findings with depression, lower serum leptin levels in women have been associated with anxiety symptoms and perceived stress [26].

Disrupted leptin signaling is also implicated in poor fetal growth as evidence suggests that growth impaired pregnancies are characterized by altered leptin physiology in cord blood [29–31], placenta [32, 33], and maternal serum [34], although reports on maternal serum have varied in designs and results [34–43]). Additionally, laboratory studies (reviewed in [44]) demonstrate that leptin plays a key role in promoting placental trophoblast proliferation and survival.

Overall it appears that leptin plays important roles in 1) the regulation of nutritional intake and energy balance, 2) the physiology of depression and anxiety, and 3) the orchestration of fetal nutritional intake and growth. This pleiotropy in leptin function may allow us to identify a subgroup of pregnancies (those complicated by depression or anxiety disorders) that may be at increased risk of poor fetal growth due to a mechanism that involves altered leptin signaling. Future research should aim to validate and further characterize the link between poor fetal growth and leptin signaling.

To our knowledge this is the first report linking placental LEPR/cg21655790 methylation levels to active maternal psychiatric disease and poor fetal growth. We are not aware of any specific evidence in the literature which suggests that a change in placental LEPR/cg21655790 methylation would alter placental leptin receptor expression levels or function in pregnancy. However there is evidence from other tissues that links altered leptin receptor methylation in to changes in leptin receptor expression and functional changes in leptin signaling. Experiments in mice suggest that a high fat diet in late gestation can result in increased methylation at CPG sites near the leptin receptor in visceral fat of the offspring and decreased leptin receptor expression (mRNA) in this same tissue [45]. These changes are tissue specific, and are not observed in skeletal muscle. Additionally, the mice exposed to this high fat late gestational diet (which appears to increase methylation at the leptin receptor gene in visceral fat) also exhibited increased food/calorie intake and faster increases in body weight in the first 20 weeks of life. Thus although we cannot cite papers that demonstrate the physiologic importance of LEPR/cg21655790 methylation in placenta, there is evidence from other tissues which suggests that methylation changes near the leptin receptor gene may have functional consequences.

We recognize that this study has both weaknesses and strengths that are worthy of mention. In terms of weaknesses, this analysis was not based on a random sample and it is possible that those who were included were in some way not ideally representative of the source population. We also note that we had limited sample sizes in the strata defined by psychiatric status and antidepressant use. Nonetheless we detected significant associations within these small strata. As in any observational study the potential for unrecognized bias or uncontrolled confounding cannot be ruled out, but the associations with maternal psychiatric disease were robust to adjustment for several potential confounders. We also note a number of strengths with our study. We were able to validate and further characterize a previously observed association that should provide insight into the etiologies of poor fetal growth. Our epidemiologic characterization then allowed us to make predictions about the biological factors that may be involved, and we were able to screen available epigenetic data to find a biological factor that met these predictions. This non-traditional strategy for DNA methylation analysis may have utility in other settings where genome-wide methylation measurements have been made in physiologically relevant tissue.

## Conclusions

In this work we validate and further characterize the association between maternal psychiatric symptoms and poor fetal growth. We found that mothers who experienced active depression or anxiety in pregnancy were more likely to deliver SGA/IUGR infants, and our follow-up epigenetic analyses suggest that altered leptin signaling may be involved in the biologic mechanisms that cause these maternal and fetal pathologies to co-occur. This work is important because depression and anxiety are very common conditions in pregnancy (about 7-20 % for depression [46, 47] and 8-15 % for anxiety [48]) and thus the pathophysiology associated with these conditions may be responsible for a large proportion of SGA births. Determining the causes of poor fetal growth is a fundamental public health goal because it is associated with both perinatal morbidity and variety of lifelong health problems such as cardiovascular disease, type II diabetes, and impaired neurocognition [49–51]. The associations reported here between psychiatric disease, leptin physiology, and poor fetal growth do not necessarily reflect causal relationships, but determining the underlying factors that link them should help to identify modifiable causes of poor fetal growth. Additional validation and characterization of these associations may eventually allow us develop interventions that can safely improve the well-being of both the mother and the developing child.

## Additional files

Additional file 1: **Supplemental Materials.** a) **Table S1.** Crude associations between clinical variables and poor fetal growth (SGA/IUGR). b) **Table S2.** Characteristics of the mother-infant pairs by maternal psychiatric status. c) An explanation of the identification of candidate loci for the epigenetic analyses including: **Table S3.** Genes with known links to psychiatric disease and poor fetal growth. **Table S4.** 27 candidate loci with measured placental DNA methylation levels. d) **Table S5.** Loci with methylation patterns that were significantly associated with poor fetal growth. e) **Table S6.** Mean methylation levels at 6 candidate loci within strata defined by psychiatric and medication status. f) **Table S7.** Mean methylation levels at the leptin receptor locus by timing of psychiatric diagnosis among those not on antidepressant medication. g) **Table S8.** Characteristics of the mother-infant pairs by psychiatric status categories used in the methylation analysis. h) **Table S9.** Methylation at the leptin receptor locus by prenatal tobacco use among those not on antidepressant medication. i) **Table S10.** Methylation at the leptin receptor locus by maternal psychiatric status excluding those with prenatal tobacco or antidepressant use. j) **Table S11.** Odds Ratios for poor fetal growth associated with methylation at LEPR/cg21655790 adjusted for prenatal tobacco use. k) **Table S12.** Characteristics of the mother-infant pairs included in the methylation data subset compared to those excluded. (DOCX 145 kb)

## Abbreviations

SGA: Small for Gestational Age
LGA: Large for Gestational Age
IUGR: Intrauterine Growth Restriction
OCD: Obsessive Compulsive Disorder
FDR: False Discovery Rate
