# Supplementary material for: Maternal psychiatric disease and epigenetic evidence suggest a common biology for poor fetal growth
Source: BMC Pregnancy Childbirth. 2015 Aug 25;15:192. doi: 10.1186/s12884-015-0627-8 (PMC4548904; doi:10.1186/s12884-015-0627-8)
Supplement: Additional file 1: — Supplemental Materials. a) Table S1. Crude associations between clinical variables and poor fetal growth (SGA/IUGR). b) Table S2. Characteristics of the mother-infant pairs by maternal psychiatric status. c) An explanation of the identification of candidate loci for the epigenetic analyses including: Table S3. Genes with known links to psychiatric disease and poor fetal growth. Table S4. 27 candidate loci with measured placental DNA methylation levels. d) Table S5. Loci with methylation patterns that were significantly associated with poor fetal growth. e) Table S6. Mean methylation levels at 6 candidate loci within strata defined by psychiatric and medication status. f) Table S7. Mean methylation levels at the leptin receptor locus by timing of psychiatric diagnosis among those not on antidepressant medication. g) Table S8. Characteristics of the mother-infant pairs by psychiatric status categories used in the methylation analysis. h) Table S9. Methylation at the leptin receptor locus by prenatal tobacco use among those not on antidepressant medication. i) Table S10. Methylation at the leptin receptor locus by maternal psychiatric status excluding those with prenatal tobacco or antidepressant use. j) Table S11. Odds Ratios for poor fetal growth associated with methylation at LEPR/cg21655790 adjusted for prenatal tobacco use. k) Table S12. Characteristics of the mother-infant pairs included in the methylation data subset compared to those excluded. (DOCX 145 kb) [file 12884_2015_627_MOESM1_ESM.docx]

**Supplemental Materials**

**Supplemental Table 1. Crude associations between clinical variables and poor fetal growth (SGA/IUGR)**

|  | **OR** | **95% CI** | **FDR adjusted p-value** |
| --- | --- | --- | --- |
| **Maternal Race** |  |  | 1.2x10^-01^ |
| White (n=260) | Reference |  |  |
| Black (n=47) | 1.61 | 0.85-3.06 |  |
| Asian (n=27) | 2.21 | 0.99-4.91 |  |
| Other race or unknown (n=115) | 1.58 | 1.00-2.51 |  |
| **Maternal Height (in cm)** | 0.96 | 0.93-0.99 | 4.0x10^-02^ |
| **Maternal Height Categories (in cm)** |  |  | 4.7x10^-02^ |
| 157.5-162.6 (n=126) | Reference |  |  |
| 137.0-157.4 (n =108) | 2.00 | 1.17-3.40 |  |
| 162.7-182.8 (n -117) | 1.03 | 0.60-1.77 |  |
| **Maternal Weight (in kg)** | 0.99 | 0.97-1.00 | 1.3x10^-01^ |
| **Maternal Weight Categories (in kg)** |  |  | 1.0x10^-02^ |
| 58.7-71.5 (n=119) | Reference |  |  |
| 37.3-58.6 (n=118) | 2.40 | 1.41-4.10 |  |
| 71.6-128.2 (n=119) | 1.13 | 0.65-1.95 |  |
| **Maternal BMI** | 0.99 | 0.95-1.03 | 6.5x10^-01^ |
| **Maternal BMI Categories** |  |  | 2.4x10^-01^ |
| 22.7-27.1 (n=116) | Reference |  |  |
| 14.8-22.6 (n=114) | 1.48 | 0.87-2.53 |  |
| 27.2-48.3 (n=115) | 0.94 | 0.54-1.62 |  |
| **Maternal Age (in years)** | 0.97 | 0.93-1.00 | 8.0x10^-02^ |
| **Maternal Age Categories (in years)** |  |  | 2.2x10^-01^ |
| 26-30 (n=148) | Reference |  |  |
| 18-25 (n=156) | 1.46 | 0.91-2.33 |  |
| 31-43 (n=145) | 0.97 | 0.59-1.58 |  |
| **Maternal Age Categories (in years)**  alternate thresholds for age [[1](#_ENREF_1), [2](#_ENREF_2)] |  |  | 5.5x10^-01^ |
| 20-34 (n=342) | Reference |  |  |
| 18-19 (n=30) | 1.04 | 0.48-2.27 |  |
| 35-43 (n=77) | 0.72 | 0.42-1.24 |  |
| **Insurance Type** |  |  | 4.0x10^-02^ |
| Private insurance (n=236) | Reference |  |  |
| Public insurance (n=205) | 1.94 | 1.30-2.89 |  |
| Military insurance (n=1) | NA | NA |  |
| Self-pay (n=7) | 1.97 | 0.43-9.06 |  |
| **Gravida (# of pregnancies)** | 0.89 | 0.77-1.02 | 1.4x10^-01^ |
| **Gravida Categories** |  |  | 1.4x10^-01^ |
| 1 (n=162) | Reference |  |  |
| 2 (n=131) | 0.62 | 0.38-1.01 |  |
| 3-11 (n=153) | 0.65 | 0.41-1.03 |  |
| **First Pregnancy, (n=162)**  alternate threshold [[1](#_ENREF_1)] | 1.57 | 1.05-2.35 | 6.6x10^-02^ |
| **Term Births (# of previous term births)** | 0.78 | 0.62-1.00 | 8.4x10^-02^ |
| **Term Birth Categories** |  |  | 7.4x10^-02^ |
| 0 (n=239) | Reference |  |  |
| 1 (n=130) | 0.55 | 0.34-0.88 |  |
| 2-4 (n=75) | 0.70 | 0.41-1.22 |  |
| **Premature Births**  **(# of previous preterm births)** | 1.15 | 0.78-1.70 | 5.5x10^-01^ |
| **Premature Birth Categories** |  |  | 1.7x10^-01^ |
| 0 (n=397) | Reference |  |  |
| 1 (n=38) | 1.99 | 1.02-3.88 |  |
| 2-5 (n=11) | 0.74 | 0.19-2.85 |  |
| **Abortions (# of previous terminations or spontaneous abortions)** | 0.92 | 0.74-1.14 | 5.2x10^-01^ |
| **Abortion Categories** |  |  | 9.7x10^-01^ |
| 0 (n=313) | Reference |  |  |
| 1 (n=80) | 0.93 | 0.55-1.56 |  |
| 2-7 (n=50) | 0.94 | 0.50-1.76 |  |
| **Live Births (# of previous live births)** | 0.85 | 0.68-1.05 | 1.9x10^-01^ |
| **Live Birth Categories** |  |  | 2.2x10^-01^ |
| 0 (n=216) | Reference |  |  |
| 1 (n=141) | 0.68 | 0.43-1.06 |  |
| 2-5 (n=87) | 0.69 | 0.41-1.18 |  |
| **Hypertension History (n=16)** | 0.86 | 0.29-2.52 | 8.4x10^-01^ |
| **Hypertension in Pregnancy (n=27)** | 2.13 | 0.98-4.66 | 1.0x10^-01^ |
| **Diabetes History (n=5)** | NA |  | 9.8x10^-01^ |
| **Diabetes in Pregnancy (n=17)** | 2.81 | 1.05-7.52 | 8.0x10^-02^ |
| **Tobacco Use History (n=59)** | 2.56 | 1.47-4.46 | 6.1x10^-03^ |
| **Tobacco Use in Pregnancy (n=48)** | 3.31 | 1.79-6.12 | 1.7x10^-03^ |
| **Alcohol History (n=15)** | 0.95 | 0.32-2.83 | 9.7x10^-01^ |
| **Alcohol in Pregnancy (n=3)** | 3.81 | 0.34-42.35 | 3.4x10^-01^ |
| **Recreational Drugs History (n=22)** | 4.39 | 1.75-11.02 | 9.7x10^-03^ |
| **Recreational Drugs in Pregnancy (n=10)** | 4.59 | 1.17-18.00 | 6.6x10^-02^ |
| **Depression History (n=60)** | 2.81 | 1.61-4.88 | 2.1x10^-03^ |
| **Depression in Pregnancy (n=46)** | 3.90 | 2.06-7.36 | 6.7x10^-04^ |
| **Anxiety History (n=38)** | 2.66 | 1.36-5.22 | 1.9x10^-02^ |
| **Anxiety Pregnancy (n=32)** | 3.15 | 1.51-6.57 | 1.1x10^-02^ |
| **OCD/PANIC History (n=8)** | 6.13 | 1.22-30.75 | 6.6x10^-02^ |
| **OCD/PANIC in Pregnancy (n=10)** | 8.23 | 1.73-39.26 | 3.0x10^-02^ |
| **Psychiatric Diagnosis History (n=74) *Depression, Anxiety, or OCD/Panic*** | 2.64 | 1.59-4.39 | 1.8x10^-03^ |
| **Psychiatric diagnosis in Pregnancy (n=58)**  ***Depression, Anxiety, or OCD/Panic*** | 3.93 | 2.21-6.98 | 1.4x10^-04^ |
| **Ever Psychiatric Diagnosis (n=76) *Depression, Anxiety, or OCD/Panic*** | 2.83 | 1.71-4.69 | 8.6x10^-04^ |
| **Female Infant (n=223)** | 0.62 | 0.42-0.92 | 4.7x10^-02^ |
| **Prenatal Vitamins (n=355)** | 0.86 | 0.53-1.39 | 5.9x10^-01^ |
| **Zoloft (n=13)** | 2.42 | 0.80-7.34 | 1.7x10^-01^ |
| **SSRI (n=18)** | 3.35 | 1.27-8.84 | 4.4x10^-02^ |
| **Atypical Antidepressant (n=5)** | 3.06 | 0.51-18.53 | 2.8x10^-01^ |
| **Any Antidepressant (n=21)** | 3.51 | 1.42-8.67 | 2.6x10^-02^ |
| **Anxiolytic (n=13)** | 3.36 | 1.08-10.46 | 7.7x10^-02^ |

**Supplemental Table 2. Characteristics of the mother-infant pairs by maternal psychiatric status**

|  | **No Depression, Anxiety, or OCD/Panic disorder in pregnancy**  **n = 381 (86.8%)^a^** | | **Depression, Anxiety, or OCD/Panic disorder in pregnancy**  **n = 58 (13.2%)^a^** | | **p-value for difference by psych status** |
| --- | --- | --- | --- | --- | --- |
|  | **Median** | **IQR** | **Median** | **IQR** |  |
| **Maternal Age in years** | 28 | 24-32 | 26 | 22-32 | 2.7x10^-01 b^ |
| **Maternal Height in cm** | 160 | 157-165 | 163 | 158-165 | 2.5x10^-01 b^ |
| **Maternal Weight in kg** | 64 | 56-75 | 63 | 58-75 | 7.8x10^-01 b^ |
| **Maternal BMI** | 24 | 22-29 | 25 | 22-29 | 7.4x10^-01 b^ |
|  |  |  |  |  |  |
|  | **Frequency** | **%** | **Frequency** | **%** |  |
| **Race** |  |  |  |  |  |
| White | 214 | 56.2 | 38 | 65.5 | 5.9x10^-01 c^ |
| Black | 40 | 10.5 | 6 | 10.3 |  |
| Asian | 25 | 6.6 | 2 | 3.5 |  |
| Other Race or Unknown | 102 | 26.8 | 12 | 20.7 |  |
| **Hypertension in pregnancy** |  |  |  |  |  |
| No | 356 | 93.7 | 56 | 96.6 | 5.6x10^-01 c^ |
| Yes | 24 | 6.3 | 2 | 3.5 |  |
| **Diabetes in pregnancy** |  |  |  |  |  |
| No | 367 | 96.6 | 55 | 94.8 | 4.6x10^-01 c^ |
| Yes | 13 | 3.4 | 3 | 5.2 |  |
| **Tobacco in pregnancy** |  |  |  |  |  |
| No | 353 | 92.9 | 40 | 69.0 | 2.3x10^-08 d^ |
| Yes | 27 | 7.1 | 18 | 31.0 |  |
| **Alcohol in pregnancy** |  |  |  |  |  |
| No | 379 | 99.5 | 57 | 98.3 | 3.5x10^-01 c^ |
| Yes | 2 | 0.5 | 1 | 1.7 |  |
| **Recreational Drugs in pregnancy** |  |  |  |  |  |
| No | 373 | 98.2 | 54 | 94.7 | 1.3x10^-01 c^ |
| Yes | 7 | 1.8 | 3 | 5.3 |  |
| **Prenatal Vitamins** |  |  |  |  |  |
| No | 73 | 19.5 | 11 | 19.0 | 9.2x10^-01 d^ |
| Yes | 301 | 80.5 | 47 | 81.0 |  |
| **Insurance** |  |  |  |  |  |
| Public | 163 | 42.8 | 37 | 63.8 | 2.0x10^-02 c^ |
| Private | 210 | 55.1 | 21 | 36.2 |  |
| Military | 1 | 0.3 | 0 | 0.0 |  |
| Self-Pay | 7 | 1.8 | 0 | 0.0 |  |
| **Antidepressant medication in pregnancy** |  |  |  |  |  |
| No | 358 | 99.7 | 36 | 65.5 | 1.8x10^-17 c^ |
| Yes | 1 | 0.3 | 19 | 34.6 |  |
| **First pregnancy**  threshold [[1](#_ENREF_1)] |  |  |  |  |  |
| No | 237 | 62.5 | 41 | 70.7 | 2.3x10^-01 d^ |
| Yes | 142 | 37.5 | 17 | 29.3 |  |
| **Maternal Age Categories (in years)**  thresholds for age [[1](#_ENREF_1), [2](#_ENREF_2)] |  |  |  |  |  |
| 18-19 | 27 | 7.1 | 1 | 1.7 | 3.3x10^-01 c^ |
| 20-34 | 289 | 75.9 | 47 | 81.0 |  |
| 35-43 | 65 | 17.1 | 10 | 17.2 |  |
| **Infant Sex** |  |  |  |  |  |
| Male | 189 | 49.6 | 32 | 55.2 | 4.3x10^-01 d^ |
| Female | 192 | 50.4 | 26 | 44.8 |  |
|  |  |  |  |  |  |
|  | **Mean** | **SD** | **Mean** | **SD** |  |
| **Pregnancy History Variables (GTPAL)** |  |  |  |  |  |
| Gravida (total # of pregnancies) | 2.25 | 1.43 | 2.74 | 1.93 | 5.5x10^-02 b^ |
| Term births (# of previous term births) | 0.67 | 0.86 | 0.68 | 0.87 | 9.1x10^-01 b^ |
| Premature births (# of previous preterm births) | 0.12 | 0.42 | 0.28 | 0.77 | 3.3x10^-02 b^ |
| Abortions (# of previous abortions) | 0.42 | 0.88 | 0.82 | 1.30 | 3.9x10^-03 b^ |
| Live births (# of previous live births) | 0.77 | 0.94 | 0.84 | 1.00 | 6.7x10^-01 b^ |

^a^ 10 were missing information on psychiatric status

^b^ Wilcoxon-Mann-Whitney test

^c^ Fisher’s Exact test

^d^ Chi Square test

Identification of candidate loci for the epigenetic analyses

We identified a recent paper that listed genes linked to poor fetal growth. [[3](#_ENREF_3)] These genes were often implicated by gene variants or differential expression patterns. Subsequent investigation of these genes lead to the identification of other genes linked to SGA/IUGR, and the hits from Banister et al [[4](#_ENREF_4)] were also evaluated. We then scanned OMIM (http://www.ncbi.nlm.nih.gov/omim) and Pubmed (http://www.ncbi.nlm.nih.gov/pubmed/) for evidence linking these genes to psychiatric disease. This process identified 11 genes with links to both SGA/IUGR and psychiatric disease. One imprinted gene, *MEG3* (Maternally Expressed Gene 3) [[5](#_ENREF_5), [6](#_ENREF_6)], was excluded because methylation measurements at imprinted genes could be unreliable on the Illumina 27K array platform, as the targeted loci may not be in the imprinting control region.

**Supplemental Table 3. Genes with known links to psychiatric disease and poor fetal growth^a^**

| **Gene** | **References and evidence linking the gene to psychiatric disease and poor fetal growth** |
| --- | --- |
| *ADCY5*  Adenylate Cyclase 5 | [[7-11](#_ENREF_7)] |
| *IGF1*  Insulin-Like Growth Factor 1 | [[12-14](#_ENREF_12)] |
| *LEKR1/CCNL1*  Leucine-, Glutamate-, and Lysine-Rich Protein 1 / Cyclin L1 | [[8](#_ENREF_8), [15-17](#_ENREF_15)] |
| *LEP*  Leptin | [[18-23](#_ENREF_18)] |
| *IGFBP1*  Insulin-like Growth Factor-Binding Protein 1 | [[24-26](#_ENREF_24)] |
| *CRH*  Corticotropin Releasing Hormone | [[27-30](#_ENREF_27)] |
| *HSD11B2*  11-Beta-Hydroxysteroid Dehydrogenase, Type II | [[25](#_ENREF_25), [31-34](#_ENREF_31)] |
| *HSD11B1*  11-Beta-Hydroxysteroid Dehydrogenase, Type I | [[25](#_ENREF_25), [35](#_ENREF_35), [36](#_ENREF_36)] |
| *RPE65*  Retinal Pigment Epithelium-Specific Protein, 65-KD | [[4](#_ENREF_4), [37](#_ENREF_37), [38](#_ENREF_38)] |
| *NFKBIZ*  Nuclear Factor of Kappa Light Chain Gene Enhancer  in B Cells Inhibitor, Zeta | [[4](#_ENREF_4), [39](#_ENREF_39), [40](#_ENREF_40)] |

^a^ Note: MEG3, Maternally Expressed Gene 3 at 14q32.2, was also identified as a candidate gene [[5](#_ENREF_5), [6](#_ENREF_6)], but it was excluded from the analysis because methylation measurements at imprinted genes may be problematic using the Illumina 27K array platform.

Then we performed texts searches for these 10 genes in the annotation file for the Human-Methylation-27 BeadChip array (Illumina Inc., San Diego, CA) and identified 27 loci with annotations that related to these genes.

**Supplemental Table 4: 27 candidate loci with measured Placental DNA methylation levels^a^**

| **Locus ID #** | **Gene Name** | **Gene Product** |
| --- | --- | --- |
| cg08636224 | FKBP5 | FK506 binding protein 5 |
| cg00862770 | FKBP5 | FK506 binding protein 5 |
| cg13384396 | ADCY5 | adenylate cyclase 5 |
| cg13878010 | ADCY5 | adenylate cyclase 5 |
| cg01305421 | IGF1 | insulin-like growth factor 1 (somatomedin C) |
| cg14568338 | IGF1R | insulin-like growth factor 1 receptor precursor |
| cg22375192 | IGF1R | insulin-like growth factor 1 receptor precursor |
| cg10691006 | MCM2^b^ | minichromosome maintenance protein 2 |
| cg15057726 | MCM2^b^ | minichromosome maintenance protein 2 |
| cg15680574 | CCNL1 | cyclin L1 |
| cg24408817 | CCNL1 | cyclin L1 |
| cg05660795 | IGFBP1 | insulin-like growth factor binding protein 1 isoform a precursor |
| cg27447599 | IGFBP1 | insulin-like growth factor binding protein 1 isoform a precursor |
| cg18640030 | CRH | corticotropin releasing hormone precursor |
| cg20329958 | CRH | corticotropin releasing hormone precursor |
| cg08789908 | HSD11B2 | hydroxysteroid (11-beta) dehydrogenase 2 |
| cg20981893 | HSD11B2 | hydroxysteroid (11-beta) dehydrogenase 2 |
| cg04732193 | HSD11B1 | 11-beta-hydroxysteroid dehydrogenase 1 |
| cg12782180 | LEP | leptin precursor |
| cg13446852 | LEPR | leptin receptor isoform 2 |
| cg21655790 | LEPR | leptin receptor isoform 2 |
| cg00177923 | LEPROTL1 | leptin receptor overlapping transcript-like 1 |
| cg18986713 | LEPROTL1 | leptin receptor overlapping transcript-like 1 |
| cg11724759 | RPE65 | retinal pigment epithelium-specific protein 65kDa |
| cg26555310 | RPE65 | retinal pigment epithelium-specific protein 65kDa |
| cg15006396 | NFKBIZ | nuclear factor of kappa light polypeptide gene enhancer in B-cells inhibitor;  zeta isoform a |
| cg26284390 | NFKBIZ | nuclear factor of kappa light polypeptide gene enhancer in B-cells inhibitor;  zeta isoform a |

^a^ information from the Illumina Human-Methylation-27 BeadChip array annotation file

(Illumina Inc., San Diego, CA)

^b^ note that the one of the annotation fields for MCM2 mentioned “cyclin-like 1”

**Supplemental Table 5. Loci with methylation patterns that were significantly associated with poor fetal growth**

| **Locus ID #** | **Gene Name** | **OR^a^** | **95% CI** | **FDR adjusted**  **p-value** |
| --- | --- | --- | --- | --- |
| cg08636224 | FKBP5 | 3.38 | 1.64-6.97 | 1.7x10^-02^ |
| cg11724759 | RPE65 | 15.79 | 4.29-58.13 | 1.8x10^-03^ |
| cg21655790 | LEPR | 0.31 | 0.14-0.67 | 2.6x10^-02^ |
| cg00177923 | LEPROTL1 | 0.14 | 0.04-0.47 | 2.0x10^-02^ |
| cg26284390 | NFKBIZ | 0.30 | 0.16-0.55 | 2.5x10^-03^ |
| cg15006396 | NFKBIZ | 0.27 | 0.12-0.63 | 2.5x10^-02^ |

^a^ Odds ratio for poor fetal growth (SGA or IUGR) associated with a one unit increase in relative methylation

**Supplemental Table 6. Mean methylation levels at 6 candidate loci within strata defined by psychiatric and medication status**

| **Gene**  **And Locus** | **No Psychiatric Diagnosis and No Antidepressant**  **(n = 159)** | **Psychiatric Diagnosis**  **and No Antidepressant**  **(n = 13)** | **Psychiatric Diagnosis and Antidepressant**  **(n=12)** |
| --- | --- | --- | --- |
|  | 59/159 (37.1%)  had SGA or IUGR | 10/13 (76.9%)  had SGA or IUGR | 9/12 (75.0%)  had SGA or IUGR |
|  | **Mean Methylation Level (Standard deviation)** | | |
| **FKBP5**  **cg08636224** | 1.00 (0.41) | 1.13 (0.41) | 0.84 (0.48) |
| **RPE65 cg11724759** | 0.38 (0.24) | 0.51 (0.26) | 0.37 (0.33) |
| **LEPR cg21655790** | -1.21 (0.39) | -1.36 (0.28) | -1.14 (0.46) |
| **LEPROTL1 cg00177923** | -3.27 (0.27) | -3.33 (0.19) | -3.18 (0.27) |
| **NFKBIZ**  **cg26284390** | -1.55 (0.52) | -1.72 (0.30) | -1.47 (0.69) |
| **NFKBIZ cg15006396** | -1.36 (0.37) | -1.48 (0.21) | -1.23 (0.42) |

**Supplemental Table 7. Mean methylation levels at the leptin receptor locus by timing of psychiatric diagnosis among those not on antidepressant medication**

| **Gene**  **And Locus** | **No History of Psychiatric Diagnosis**  **(n = 151)** | **History of Psychiatric Diagnosis but no active diagnosis**  **(n = 8)** | **Active Psychiatric Diagnosis**  **(n=13)** |
| --- | --- | --- | --- |
|  | 57/151 (37.8%)  had SGA or IUGR | 2/8 (25.0%)  had SGA or IUGR | 10/13 (76.9%)  had SGA or IUGR |
|  | **Mean Methylation Level (Standard deviation)** | | |
| **LEPR^a^ cg21655790** | -1.22 (0.40) | -0.98 (0.32) | -1.36 (0.28) |

**Supplemental Table 8. Characteristics of the mother-infant pairs by psychiatric status categories used in the methylation analysis**

|  | **No Psychiatric Diagnosis and No Antidepressant**  **(n = 159)** | | **Psychiatric Diagnosis**  **and No Antidepressant**  **(n = 13)** | | **Psychiatric Diagnosis and Antidepressant**  **(n=12)** | | **p-value**  **for difference between categories** |
| --- | --- | --- | --- | --- | --- | --- | --- |
|  | **Median** | **IQR** | **Median** | **IQR** | **Median** | **IQR** |  |
| **Maternal Age in years** | 28 | 23-31 | 24 | 21-36 | 28 | 21-31 | 9.0x10^-01 a^ |
| **Maternal Height in cm** | 160 | 155-165 | 163 | 160-164 | 160 | 157-166 | 9.0 x10^-01 a^ |
| **Maternal Weight in kg** | 63 | 55-75 | 61 | 54-67 | 65 | 53-83 | 8.2 x10^-01 a^ |
| **Maternal BMI** | 24 | 22-28 | 24 | 21-25 | 26 | 20-29 | 8.5 x10^-01 a^ |
|  |  |  |  |  |  |  |  |
|  | **Frequency** | **%** | **Frequency** | **%** | **Frequency** | **%** |  |
| **Race** |  |  |  |  |  |  | 5.8x10^-01 b^ |
| White | 78 | 49.1 | 7 | 53.9 | 8 | 66.7 |  |
| Black | 18 | 11.3 | 0 | 0.0 | 2 | 16.7 |  |
| Asian | 15 | 9.4 | 0 | 0.0 | 0 | 0.0 |  |
| Other Race or Unknown | 48 | 30.2 | 6 | 46.2 | 2 | 16.7 |  |
| **Hypertension in pregnancy** |  |  |  |  |  |  | 1.0 ^b^ |
| No | 150 | 94.9 | 13 | 100.0 | 12 | 100.0 |  |
| Yes | 8 | 5.1 | 0 | 0.0 | 0 | 0.0 |  |
| **Diabetes in pregnancy** |  |  |  |  |  |  | 7.0 x10^-01 b^ |
| No | 151 | 95.6 | 12 | 92.3 | 12 | 100.0 |  |
| Yes | 7 | 4.4 | 1 | 7.7 | 0 | 0.0 |  |
| **Tobacco in pregnancy** |  |  |  |  |  |  | 3.2 x10^-04 bc^ |
| No | 148 | 93.1 | 8 | 61.5 | 8 | 66.7 |  |
| Yes | 11 | 6.9 | 5 | 38.5 | 4 | 33.3 |  |
| **Alcohol in pregnancy** |  |  |  |  |  |  | 1.0 ^b^ |
| No | 158 | 99.4 | 13 | 100.0 | 12 | 100.0 |  |
| Yes | 1 | 0.6 | 0 | 0.0 | 0 | 0.0 |  |
| **Recreational Drugs in pregnancy** |  |  |  |  |  |  | 1.4 x10^-01 b^ |
| No | 155 | 98.1 | 12 | 92.3 | 11 | 91.7 |  |
| Yes | 3 | 1.9 | 1 | 7.7 | 1 | 8.3 |  |
| **Prenatal Vitamins** |  |  |  |  |  |  | 9.1 x10^-01 b^ |
| No | 25 | 15.7 | 2 | 15.4 | 1 | 8.3 |  |
| Yes | 134 | 84.3 | 11 | 84.6 | 11 | 91.7 |  |
| **Insurance** |  |  |  |  |  |  | 1.3 x10^-01 b^ |
| Public | 70 | 44.0 | 10 | 76.9 | 8 | 66.7 |  |
| Private | 85 | 53.5 | 3 | 23.1 | 4 | 33.3 |  |
| Military | 0 | 0.0 | 0 | 0.0 | 0 | 0.0 |  |
| Self-Pay | 4 | 2.5 | 0 | 0.0 | 0 | 0.0 |  |
| **First pregnancy**  threshold [[1](#_ENREF_1)] |  |  |  |  |  |  | 1.0 ^b^ |
| No | 105 | 66.0 | 9 | 69.2 | 8 | 66.7 |  |
| Yes | 54 | 34.0 | 4 | 30.8 | 4 | 33.3 |  |
| **Maternal Age Categories (in years)**  thresholds for age [[1](#_ENREF_1), [2](#_ENREF_2)] |  |  |  |  |  |  | 5.8 x10^-01 b^ |
| 18-19 | 10 | 6.3 | 0 | 0.0 | 0 | 0.0 |  |
| 20-34 | 125 | 78.6 | 9 | 69.2 | 10 | 83.3 |  |
| 35-43 | 24 | 15.1 | 4 | 30.8 | 2 | 16.7 |  |
| **Infant Sex** |  |  |  |  |  |  | 2.0 x10^-01 b^ |
| Male | 76 | 47.8 | 9 | 69.2 | 4 | 33.3 |  |
| Female | 83 | 52.2 | 4 | 30.8 | 8 | 66.7 |  |
|  |  |  |  |  |  |  |  |
|  | **Mean** | **SD** | **Mean** | **SD** | **Mean** | **SD** |  |
| **Pregnancy History Variables (GTPAL)** |  |  |  |  |  |  |  |
| Gravida (total # of  pregnancies) | 2.17 | 1.21 | 2.54 | 1.45 | 2.83 | 1.80 | 3.2x10^-01 a^ |
| Term births (# of  previous term births) | 0.62 | 0.79 | 0.54 | 0.66 | 0.36 | 0.67 | 5.4 x10^-01 a^ |
| Premature births (# of  previous preterm births) | 0.16 | 0.50 | 0.23 | 0.44 | 0.75 | 1.48 | 6.7 x10^-02 a^ |
| Abortions (# of  previous abortions) | 0.36 | 0.69 | 0.85 | 1.07 | 0.82 | 0.87 | 1.9 x10^-02 ad^ |
| Live births (# of  previous live births) | 0.73 | 0.85 | 0.77 | 0.83 | 0.55 | 0.82 | 7.0 x10^-01 a^ |

^a^ Kruskal-Wallis test

^b^ Fisher’s Exact test

^c^ When compared to those without psychiatric diagnoses, the frequency of tobacco use was higher among those with un-medicated psychiatric diagnoses (Fisher’s Exact test p=3.0x10^-3^) and those with medicated psychiatric diagnoses (Fisher’s Exact test p=1.3x10^-2^). There was no evidence for a difference in tobacco use between those with medicated and un-medicated psychiatric diagnoses (Fisher’s Exact test p=1.0).

^d^ When compared to those without psychiatric diagnoses, the history of abortion was more frequent among those with un-medicated psychiatric diagnoses (Wilcoxon test p=4.1x10^-2^) and those with medicated psychiatric diagnoses (Wilcoxon test p=3.6x10^-2^). There was no evidence for a difference in abortion history between those with medicated and un-medicated psychiatric diagnoses (Wilcoxon test p=9.3x10^-1^).

**Discussion of Supplemental Table 8:**

**Factors in addition to methylation at LEPR/cg21655790 that differ by maternal psychiatric status**

We found that those with psychiatric disease in pregnancy (both medicated and un-medicated) were more likely to use tobacco than those without active psychiatric disease. Our initial findings indicated a significant association between psychiatric disease in pregnancy and low methylation at LEPR/cg21655790, but based on these new results we also investigated the relationship between methylation at LEPR/cg21655790 and prenatal tobacco use. We found a trend among un-medicated participants suggesting that prenatal tobacco use may also be associated low methylation levels at LEPR/cg21655790 (Fisher’s exact test p=1.3x10^-1^, see Supplemental Table 9). However, the association between un-medicated psychiatric disease and decreased methylation at LEPR/cg21655790 is still significant when those who used tobacco in pregnancy are excluded from the analysis (p=3.3x10^-2^, Supplemental Table 10). This indicates that prenatal tobacco use does not explain the association between decreased LEPR/cg21655790 methylation and psychiatric disease. Additionally, the association between decreased methylation at LEPR/cg21655790 and poor fetal growth still significant in a logistic regression model adjusted for prenatal tobacco use (p=8.5x10^-3^, Supplemental Table 11). This indicates that prenatal tobacco use also does not explain the association between decreased methylation at LEPR/cg21655790 and poor fetal growth. Furthermore, we already know form Table 2a in the main text that the association between prenatal psychiatric disease and poor fetal growth (SGA/IUGR) is still significant after adjusting for tobacco use in pregnancy. This suggests that prenatal tobacco use also does not explain the association between active psychiatric disease and poor fetal growth. Therefore the links between psychiatric disease, poor fetal growth and decreased methylation at the LEPR locus are not explained by tobacco use.

Note that women with psychiatric disease (medicated and un-medicated) were also more likely to have a history of abortion (spontaneous or otherwise). Because the abnormal physiology associated with decreased placental methylation at LEPR/cg21655790 may be responsible for a variety of adverse pregnancy outcomes (abortion, poor fetal growth, maternal psychiatric symptoms, etc) we would not consider abortion history to confound the association between placental methylation at LEPR/cg21655790 and either psychiatric disease or SGA/IUGR (see Howards et al 2007 [[41](#_ENREF_41)] for further discussion).

**Supplemental Table 9. Methylation at the leptin receptor locus by prenatal tobacco use among those not on antidepressant medication**

| **Gene**  **And Locus** | **Methylation**  **(adjusted Beta)** | **No Prenatal Tobacco Use**  **(n = 159)** | **Prenatal**  **Tobacco Use**  **(n = 17)** | **p-value**  **(Fisher’s exact test)** |
| --- | --- | --- | --- | --- |
| **LEPR^a^ cg21655790** | <= Median | 79 (49.7%) | 12 (70.6%) | 1.3 x 10^-1^ |
|  | > Median | 80 (50.3%) | 5 (29.4%) |  |

**Supplemental Table 10. Methylation at the leptin receptor locus by maternal psychiatric status excluding those with prenatal tobacco or antidepressant use**

| **Gene**  **And Locus** | **Methylation**  **(adjusted Beta)** | **No Psychiatric Diagnosis**  **- No antidepressant or tobacco use**  **(n = 148)** | **Psychiatric Diagnosis**  **- No antidepressant or tobacco use**  **(n = 8)** | **p-value**  **(Fisher’s exact test)** |
| --- | --- | --- | --- | --- |
| **LEPR^a^ cg21655790** | <= Median | 70 (47.3%) | 7 (87.5%) | 3.3 x 10^-2^ |
|  | > Median | 78 (52.7%) | 1 (12.5%) |  |

**Supplemental Table 11. Odds Ratios for poor fetal growth associated with methylation at LEPR/cg21655790 adjusted for prenatal tobacco use**

|  | **OR** | **95% CI** | **p-value** |
| --- | --- | --- | --- |
| **Unadjusted model** |  |  |  |
| Methylation at LEPR/cg21655790 | 0.31^a^ | 0.14-0.67 | 2.9x10^-3^ |
|  |  |  |  |
| **Prenatal tobacco use adjusted model** |  |  |  |
| Methylation at LEPR/cg21655790 | 0.34^b^ | 0.15-0.76 | 8.5x10^-3^ |
| Prenatal tobacco use | 5.96^c^ | 1.91-18.63 | 2.1x10^-3^ |

^a^ Odds ratio for poor fetal growth (SGA or IUGR) associated with a one unit increase in relative methylation

^b^ Odds ratio for poor fetal growth (SGA or IUGR) associated with a one unit increase in relative methylation after

adjusting for the prenatal tobacco use

^c^ Odds ratio for poor fetal growth associated with prenatal tobacco use after adjusting for methylation at

LEPR/cg21655790

**Supplemental Table 12. Characteristics of the mother-infant pairs included in the methylation data subset compared to those excluded**

|  | **No Methylation Data**  **(excluded from the methylation analyses)**  **n = 252** | | **Methylation Data**  **(included in the methylation analyses)**  **n = 197** | | **p-value for difference** |
| --- | --- | --- | --- | --- | --- |
|  | **Median** | **IQR** | **Median** | **IQR** |  |
| **Maternal Age in years** | 28 | 24-32 | 28 | 23-32 | 5.4x10^-01 a^ |
| **Maternal Height in cm** | 160 | 157-165 | 160 | 155-165 | 9.1x10^-01 a^ |
| **Maternal Weight in kg** | 64 | 57-75 | 62 | 55-75 | 2.5x10^-01 a^ |
| **Maternal BMI** | 25 | 22-29 | 24 | 22-29 | 7.6x10^-02 a^ |
|  |  |  |  |  |  |
|  | **Frequency** | **%** | **Frequency** | **%** |  |
| **Race** |  |  |  |  |  |
| White | 158 | 62.7 | 102 | 51.8 | 7.5x10^-02 b^ |
| Black | 26 | 10.3 | 21 | 10.7 |  |
| Asian | 11 | 4.4 | 16 | 8.1 |  |
| Other Race or Unknown | 57 | 22.6 | 58 | 29.4 |  |
| **Hypertension in pregnancy** |  |  |  |  |  |
| No | 234 | 93.2 | 186 | 94.9 | 4.6x10^-01 b^ |
| Yes | 17 | 6.8 | 10 | 5.1 |  |
| **Diabetes in pregnancy** |  |  |  |  |  |
| No | 243 | 96.8 | 187 | 95.4 | 4.4x10^-01 b^ |
| Yes | 8 | 3.2 | 9 | 4.6 |  |
| **Tobacco in pregnancy** |  |  |  |  |  |
| No | 224 | 89.6 | 175 | 88.8 | 7.9x10^-01 b^ |
| Yes | 26 | 10.4 | 22 | 11.2 |  |
| **Alcohol in pregnancy** |  |  |  |  |  |
| No | 249 | 99.2 | 196 | 99.5 | 1.0 ^c^ |
| Yes | 2 | 0.8 | 1 | 0.5 |  |
| **Recreational Drugs in pregnancy** |  |  |  |  |  |
| No | 246 | 98.4 | 190 | 96.9 | 3.5x10^-01 c^ |
| Yes | 4 | 1.6 | 6 | 3.1 |  |
| **Prenatal Vitamins** |  |  |  |  |  |
| No | 50 | 20.4 | 37 | 18.8 | 6.7x10^-01 b^ |
| Yes | 195 | 79.6 | 160 | 81.2 |  |
| **Insurance** |  |  |  |  |  |
| Public | 113 | 44.8 | 92 | 46.7 | 3.6x10^-01 c^ |
| Private | 136 | 54.0 | 100 | 50.8 |  |
| Military | 1 | 0.4 | 0 | 0.0 |  |
| Self-Pay | 2 | 0.8 | 5 | 2.5 |  |
| **Psychiatric diagnosis in pregnancy** |  |  |  |  |  |
| No | 215 | 87.0 | 166 | 86.46 | 8.6x10^-01 b^ |
| Yes | 32 | 13.0 | 26 | 13.54 |  |
| **Antidepressant medication in pregnancy** |  |  |  |  |  |
| No | 225 | 96.6 | 176 | 93.1 | 1.1x10^-01 b^ |
| Yes | 8 | 3.4 | 13 | 6.9 |  |
| **First pregnancy**  threshold [[1](#_ENREF_1)] |  |  |  |  |  |
| No | 153 | 61.5 | 131 | 66.5 | 2.7x10^-01 b^ |
| Yes | 96 | 38.6 | 66 | 33.5 |  |
| **Maternal Age Categories (in years)**  thresholds for age [[1](#_ENREF_1), [2](#_ENREF_2)] |  |  |  |  |  |
| 18-19 | 19 | 7.5 | 11 | 5.6 | 6.1x10^-01 b^ |
| 20-34 | 188 | 74.6 | 154 | 78.2 |  |
| 35-43 | 45 | 17.9 | 32 | 16.2 |  |
| **Infant Sex** |  |  |  |  |  |
| Male | 132 | 52.4 | 94 | 47.7 | 3.3x10^-01 b^ |
| Female | 120 | 47.6 | 103 | 52.3 |  |
|  |  |  |  |  |  |
|  | **Mean** | **SD** | **Mean** | **SD** |  |
| **Pregnancy History Variables (GTPAL)** |  |  |  |  |  |
| Gravida  (total # of pregnancies) | 2.38 | 1.68 | 2.24 | 1.27 | 8.8x10^-01 a^ |
| Term births  (# of previous term births) | 0.73 | 0.92 | 0.60 | 0.77 | 2.2x10^-01 a^ |
| Premature births  (# of previous preterm births) | 0.10 | 0.35 | 0.20 | 0.61 | 5.0x10^-02 a^ |
| Abortions  (# of previous abortions) | 0.52 | 1.09 | 0.43 | 0.74 | 6.3x10^-01 a^ |
| Live births  (# of previous live births) | 0.83 | 1.02 | 0.72 | 0.83 | 6.2x10^-01 a^ |

^a^ Wilcoxon-Mann-Whitney test

^b^ Chi-Square test

^c^ Fisher’s Exact test

**References**

1. McCowan L, Horgan RP: **Risk factors for small for gestational age infants**. *Best practice & research Clinical obstetrics & gynaecology* 2009, **23**(6):779-793.

2. Khashan AS, Baker PN, Kenny LC: **Preterm birth and reduced birthweight in first and second teenage pregnancies: a register-based cohort study**. *BMC pregnancy and childbirth* 2010, **10**:36.

3. Ishida M, Moore GE: **The role of imprinted genes in humans**. *Molecular aspects of medicine* 2012.

4. Banister CE, Koestler DC, Maccani MA, Padbury JF, Houseman EA, Marsit CJ: **Infant growth restriction is associated with distinct patterns of DNA methylation in human placentas**. *Epigenetics : official journal of the DNA Methylation Society* 2011, **6**(7):920-927.

5. Takahashi N, Okamoto A, Kobayashi R, Shirai M, Obata Y, Ogawa H, Sotomaru Y, Kono T: **Deletion of Gtl2, imprinted non-coding RNA, with its differentially methylated region induces lethal parent-origin-dependent defects in mice**. *Human molecular genetics* 2009, **18**(10):1879-1888.

6. Liu Y, Murphy SK, Murtha AP, Fuemmeler BF, Schildkraut J, Huang Z, Overcash F, Kurtzberg J, Jirtle R, Iversen ES *et al*: **Depression in pregnancy, infant birth weight and DNA methylation of imprint regulatory elements**. *Epigenetics : official journal of the DNA Methylation Society* 2012, **7**(7):735-746.

7. **Fasting Plasma Glucose Level Quantitative Trait Locus 6; FGQTL6, and Birth Weight Quantitative Trait Locus 3; BWQTL3, Included, OMIM entry** [<http://omim.org/entry/613460>]

8. Freathy RM, Mook-Kanamori DO, Sovio U, Prokopenko I, Timpson NJ, Berry DJ, Warrington NM, Widen E, Hottenga JJ, Kaakinen M *et al*: **Variants in ADCY5 and near CCNL1 are associated with fetal growth and birth weight**. *Nature genetics* 2010, **42**(5):430-435.

9. Horikoshi M, Yaghootkar H, Mook-Kanamori DO, Sovio U, Taal HR, Hennig BJ, Bradfield JP, St Pourcain B, Evans DM, Charoen P *et al*: **New loci associated with birth weight identify genetic links between intrauterine growth and adult height and metabolism**. *Nature genetics* 2013, **45**(1):76-82.

10. Otsuki K, Uchida S, Wakabayashi Y, Matsubara T, Hobara T, Funato H, Watanabe Y: **Aberrant REST-mediated transcriptional regulation in major depressive disorder**. *Journal of psychiatric research* 2010, **44**(6):378-384.

11. Procopio DO, Saba LM, Walter H, Lesch O, Skala K, Schlaff G, Vanderlinden L, Clapp P, Hoffman PL, Tabakoff B: **Genetic markers of comorbid depression and alcoholism in women**. *Alcoholism, clinical and experimental research* 2013, **37**(6):896-904.

12. **Insulin-Like Growth Factor I; IGF1, OMIM entry** [<http://omim.org/entry/147440>]

13. Vaessen N, Janssen JA, Heutink P, Hofman A, Lamberts SW, Oostra BA, Pols HA, van Duijn CM: **Association between genetic variation in the gene for insulin-like growth factor-I and low birthweight**. *Lancet* 2002, **359**(9311):1036-1037.

14. Mitschelen M, Yan H, Farley JA, Warrington JP, Han S, Herenu CB, Csiszar A, Ungvari Z, Bailey-Downs LC, Bass CE *et al*: **Long-term deficiency of circulating and hippocampal insulin-like growth factor I induces depressive behavior in adult mice: a potential model of geriatric depression**. *Neuroscience* 2011, **185**:50-60.

15. **Birth Weight Quantitative Trait Locus 2; BWQTL2, OMIM entry** [<http://omim.org/entry/613459>]

16. Andersson EA, Harder MN, Pilgaard K, Pisinger C, Stancakova A, Kuusisto J, Grarup N, Faerch K, Poulsen P, Witte DR *et al*: **The birth weight lowering C-allele of rs900400 near LEKR1 and CCNL1 associates with elevated insulin release following an oral glucose challenge**. *PloS one* 2011, **6**(11):e27096.

17. Ali Khan A, Rodriguez A, Sebert S, Kaakinen M, Cauchi S, Froguel P, Hartikainen AL, Pouta A, Jarvelin MR: **The interplay of variants near LEKR and CCNL1 and social stress in relation to birth size**. *PloS one* 2012, **7**(6):e38216.

18. **Leptin; LEP, OMIM entry** [<http://omim.org/entry/164160>]

19. Karakosta P, Chatzi L, Plana E, Margioris A, Castanas E, Kogevinas M: **Leptin levels in cord blood and anthropometric measures at birth: a systematic review and meta-analysis**. *Paediatric and perinatal epidemiology* 2011, **25**(2):150-163.

20. Lu XY: **The leptin hypothesis of depression: a potential link between mood disorders and obesity?** *Current opinion in pharmacology* 2007, **7**(6):648-652.

21. Zeman M, Jirak R, Jachymova M, Vecka M, Tvrzicka E, Zak A: **Leptin, adiponectin, leptin to adiponectin ratio and insulin resistance in depressive women**. *Neuro endocrinology letters* 2009, **30**(3):387-395.

22. Morris AA, Ahmed Y, Stoyanova N, Hooper WC, De Staerke C, Gibbons G, Quyyumi A, Vaccarino V: **The association between depression and leptin is mediated by adiposity**. *Psychosomatic medicine* 2012, **74**(5):483-488.

23. **Leptin Receptor; LEPR, OMIM entry** [<http://omim.org/entry/601007>]

24. **Insulin-Like Growth Factor-Binding Protein 1; IGFBP1, OMIM entry** [<http://omim.org/entry/146730>]

25. Bomba M, Gambera A, Bonini L, Peroni M, Neri F, Scagliola P, Nacinovich R: **Endocrine profiles and neuropsychologic correlates of functional hypothalamic amenorrhea in adolescents**. *Fertility and sterility* 2007, **87**(4):876-885.

26. Okamoto A, Endo H, Kalionis B, Shinya M, Saito M, Nikaido T, Tanaka T: **IGFBP1 and Follistatin-like 3 genes are significantly up-regulated in expression profiles of the IUGR placenta**. *Placenta* 2006, **27**(2-3):317-321.

27. **Corticotropin-Releasing Hormone; CRH, OMIM entry** [<http://omim.org/entry/122560>]

28. Whitehead CL, Walker SP, Ye L, Mendis S, Kaitu'u-Lino TJ, Lappas M, Tong S: **Placental specific mRNA in the maternal circulation are globally dysregulated in pregnancies complicated by fetal growth restriction**. *The Journal of clinical endocrinology and metabolism* 2013, **98**(3):E429-436.

29. Thomson M: **The physiological roles of placental corticotropin releasing hormone in pregnancy and childbirth**. *Journal of physiology and biochemistry* 2013, **69**(3):559-573.

30. Bao AM, Swaab DF: **Corticotropin-releasing hormone and arginine vasopressin in depression focus on the human postmortem hypothalamus**. *Vitamins and hormones* 2010, **82**:339-365.

31. **11-Beta-Hydroxysteroid Dehydrogenase, Type II; HSD11B2 , OMIM entry** [<http://omim.org/entry/614232>]

32. Marsit CJ, Maccani MA, Padbury JF, Lester BM: **Placental 11-beta hydroxysteroid dehydrogenase methylation is associated with newborn growth and a measure of neurobehavioral outcome**. *PloS one* 2012, **7**(3):e33794.

33. Jensen Pena C, Monk C, Champagne FA: **Epigenetic effects of prenatal stress on 11beta-hydroxysteroid dehydrogenase-2 in the placenta and fetal brain**. *PloS one* 2012, **7**(6):e39791.

34. Ponder KL, Salisbury A, McGonnigal B, Laliberte A, Lester B, Padbury JF: **Maternal depression and anxiety are associated with altered gene expression in the human placenta without modification by antidepressant use: implications for fetal programming**. *Developmental psychobiology* 2011, **53**(7):711-723.

35. **11-Beta-Hydroxysteroid Dehydrogenase, Type I; HSD11B1 , OMIM entry** [<http://omim.org/entry/600713>]

36. Alfaidy N, Li W, MacIntosh T, Yang K, Challis J: **Late gestation increase in 11beta-hydroxysteroid dehydrogenase 1 expression in human fetal membranes: a novel intrauterine source of cortisol**. *The Journal of clinical endocrinology and metabolism* 2003, **88**(10):5033-5038.

37. **Retinal Pigment Epithelium-Specific Protein, 65-KD; RPE65, OMIM entry** [<http://www.omim.org/entry/180069>]

38. Takahashi Y, Moiseyev G, Chen Y, Farjo K, Nikolaeva O, Ma JX: **An enzymatic mechanism for generating the precursor of endogenous 13-cis retinoic acid in the brain**. *The FEBS journal* 2011, **278**(6):973-987.

39. **Nuclear Factor of Kappa Light Chain Gene Enhancer in B Cells Inhibitor, Zeta; NFKBIZ, OMIM entry** [<http://omim.org/entry/608004>]

40. Savitz J, Frank MB, Victor T, Bebak M, Marino JH, Bellgowan PS, McKinney BA, Bodurka J, Kent Teague T, Drevets WC: **Inflammation and neurological disease-related genes are differentially expressed in depressed patients with mood disorders and correlate with morphometric and functional imaging abnormalities**. *Brain, behavior, and immunity* 2013, **31**:161-171.

41. Howards PP, Schisterman EF, Heagerty PJ: **Potential confounding by exposure history and prior outcomes: an example from perinatal epidemiology**. *Epidemiology (Cambridge, Mass)* 2007, **18**(5):544-551.
